# Assessing the performance of remotely-sensed flooding indicators and their potential contribution to early warning for leptospirosis in Cambodia

**DOI:** 10.1371/journal.pone.0181044

**Published:** 2017-07-13

**Authors:** Julia Ledien, Sopheak Sorn, Sopheak Hem, Rekol Huy, Philippe Buchy, Arnaud Tarantola, Julien Cappelle

**Affiliations:** 1 Epidemiology and Public Health Unit, Institut Pasteur du Cambodge, Phnom Penh, Cambodia; 2 Medical Biology Unit, Institut Pasteur du Cambodge, Phnom Penh, Cambodia; 3 Centre National de Malariologie (CNM), Phnom Penh, Cambodia; 4 GlaxoSmithKline, Vaccines R&D, Singapore, Singapore; 5 Epidemiology unit, Institut Pasteur de Nouvelle-Calédonie, 11 rue Paul Doumer, Nouméa, New Caledonia; 6 CIRAD-ES, UPR AGIRs, Montpellier, France; 7 UMR EPIA, INRA, VetAgro Sup, Univ Lyon, Marcy-l'étoile, France; Bristol University/Remote Sensing Solutions Inc., UNITED STATES

## Abstract

Remote sensing can contribute to early warning for diseases with environmental drivers, such as flooding for leptospirosis. In this study we assessed whether and which remotely-sensed flooding indicator could be used in Cambodia to study any disease for which flooding has already been identified as an important driver, using leptospirosis as a case study. The performance of six potential flooding indicators was assessed by ground truthing. The Modified Normalized Difference Water Index (MNDWI) was used to estimate the Risk Ratio (RR) of being infected by leptospirosis when exposed to floods it detected, in particular during the rainy season. Chi-square tests were also calculated. Another variable—the time elapsed since the first flooding of the year—was created using MNDWI values and was also included as explanatory variable in a generalized linear model (GLM) and in a boosted regression tree model (BRT) of leptospirosis infections, along with other explanatory variables. Interestingly, MNDWI thresholds for both detecting water and predicting the risk of leptospirosis seroconversion were independently evaluated at -0.3. Value of MNDWI greater than -0.3 was significantly related to leptospirosis infection (RR = 1.61 [1.10–1.52]; χ2 = 5.64, p-value = 0.02, especially during the rainy season (RR = 2.03 [1.25–3.28]; χ2 = 8.15, p-value = 0.004). Time since the first flooding of the year was a significant risk factor in our GLM model (p-value = 0.042). These results suggest that MNDWI may be useful as a risk indicator in an early warning remote sensing tool for flood-driven diseases like leptospirosis in South East Asia.

## 1. Introduction

Remote sensing provides a large variety of indicators that can inform Public Health especially when diseases have environmental drivers [1]. Remotely-sensed environmental indicators can help to understand the epidemiology of such diseases, predict health risks and improve timely and targeted response to outbreaks. Models using remote sensing data can for example be used as an early warning tool when changes in environmental indicators have been shown to predict an outbreak [2,3]. After the launch of Landsat 1 in the 70’s and the development of earth observation systems in the 80’s and the 90’s, several environmental indicators produced from satellite images were used for health studies, especially for the surveillance of vector-borne diseases which usually have strong environmental drivers [4]. Remotely-sensed data were used to map different factors such as vegetation, deforestation, flooding or urban features and infer the associated risk of disease, promising the development of early warning systems for major health threats such as Rift Valley Fever or malaria [3–7].

Water-borne diseases are by definition driven by strong environmental factors. The monitoring of environmental indicators related to water can help assess the risk associated with these diseases [8]. Cholera was one of the first disease for which the use of remote sensing drastically improved the knowledge about the epidemiology of the disease and the capacity to anticipate out breaks [2]. Remotely-sensed data—including sea surface temperature and sea surface height—were used to infer the presence of the bacterium responsible for the disease [2]. This demonstrated the influence of climate on cholera outbreaks and allowed for the development of an early warning system [2]. The impact of climate change on infectious diseases in general is uncertain but satellite imaging could help in mounting an effective response [9]. Many remotely-sensed indicators have been used to study waterborne diseases whether by mapping water bodies and flooded areas or by trying to characterize different water variables of interest for disease transmission [10]. Selecting relevant indicators is a crucial step and an assessment of the performance of the different potential indicators should initiate research, especially in new study regions [11]. Despite its potential to improve the surveillance of waterborne diseases, remote sensing is still seldom used by health specialists and should be better promoted for the development of monitoring and early warning system by regional or national health authorities [8].

Leptospirosis is a worldwide bacterial disease caused by a spirochete of the genus *Leptospira*. Considered as a non-specific anthropozoonosis, leptospirosis may be transmitted by many wild and domestic species (rodents, insectivores, horses, cattle, dogs and pigs) [12]. This makes leptospirosis difficult to control in human and animal population. The incubation period ranges from 2 to 30 days after the onset of symptoms. The immune response is detectable only after 7 to 10 days [13]. Some symptoms are similar to other infections such as influenza, meningitis, hepatitis or dengue fever, which explains why clinical diagnosis is difficult and contributes to the underreporting of leptospirosis. It has been estimated that 90% of cases of leptospirosis were asymptomatic or mild [12]. Ten percent of leptospirosis infections are termed severe. In such cases, symptoms may progress to pulmonary hemorrhage, kidney or liver failure or coma [14–16]. The global case-fatality rate (CFR) for severe leptospirosis is estimated at 22% and can be greater than 70% for Weil Disease [17]. The CFR varies greatly, depending on available medical facilities [18]. It is estimated that 1,030,000 cases and 58,900 deaths are caused by leptospirosis each year, a mortality comparable to melanoma or rabies [17]. with an estimated 2.9 million Disability-Adjusted Life Year (DALYs) lost per annum [19].

In South East Asia, the annual incidence of leptospirosis is estimated at 10–100 cases per 100,000 people [20]. A recent study estimated the incidence of leptospirosis in the region at 55.54, 95% CI 20.32–99.53 per 100 000 population [17]. In Thailand, an annual incidence of 23.7 per 100,000 persons was observed in 2000. Most of the cases (90%) were reported from flooded areas [20]. In Viet Nam, a March 2003 study found a prevalence of 11% in children aged 7 years with an annual seroconversion rate of 1.5% [21]. In Lao PDR, the seroprevalence was estimated at 23.9% in the rural population aged 15 or above [22].

In Cambodia, a study estimated the annual incidence equivalent to half that of dengue, at 12.9 per 100,000 inhabitants aged below 20 years in Kampong Cham province during the 2007–2009 rainy seasons [23]. Another, hospital-based study of suspected leptospirosis cases found a seroprevalence of 29.0%; 14.4% with serological evidence of recent infection and 15.5% with evidence of previous leptospirosis infection [24]. More recently, a study on undifferentiated fevers in rural Cambodian health centers found a seroprevalence for leptospirosis of 20.8% [25]. Studies implemented in rodents in several provinces of the country showed that they were more likely to be infected by leptospirosis during the rainy season (May to December) and the prevalence were higher in rodents trapped in rice fields, and other flooded areas, forest areas or recently cleared culture areas [26].

The most common risk factors of leptospirosis infections are occupational (farmer, sewer worker), recreational (canoeing, swimming), cultural factors (bathing in rivers, taming animals, the presence of pets in the home) and socioeconomic factors (poverty, lack of sanitation) [13]. Living in a rural area, being a male or being exposed to flooding is also associated with a higher risk of infection [13,17,27].Outbreaks are mostly observed during the rainy season [28] and it seems that, in some places, flooding is the direct cause of the outbreak and not only a risk-factor [27]. Many leptospirosis outbreaks have been reported after extreme climatic events around the World [13]. The use of environmental data has proved useful in modeling the risk of leptospirosis. On Samoa Island, a study showed the importance of altitude and some other environmental variables to explain a rise in leptospirosis cases [29]. In Thailand, rainfall and temperature were the core of a time-series model on the number of leptospirosis cases [28]. The strong link between leptospirosis outbreaks and extraordinary climatic events is explained by the capacity for *Leptospira* bacteria to accumulate in the humid soil [30] which come in contact with animals and humans in case of flooding [13].

The main objective of this study is to assess if and which remotely-sensed flooding indicator could be used in Cambodia to study any disease for which flooding has already been identified as an important driver. In this paper we use leptospirosis as a case study, assuming that flooding is an important driver of leptospirosis in Cambodia as shown by Ivanova and al. for rodents [26], and as demonstrated in India, Lao PDR, Indonesia, Italy, Guyana, Nicaragua, Puerto Rico, New Caledonia, Australia and USA for Humans [13,22,31,32]. We do not explore an epidemiological mechanism involving flooding for leptospirosis but rather the possibility for national and local health authorities to easily use a remotely-sensed flooding indicator as an early warning tool for flood-driven diseases like leptospirosis. Specifically, we (i) assess the performance of various remotely-sensed indicators to detect flooded areas, select the best one and (ii) evaluate its potential use in predicting the distribution of human leptospirosis infections at local level in Kampong Cham province, Cambodia.

## 2. Material and method

### 2.1. Study area, children cohort and leptospirosis data

A cohort of villagers aged below 20 were followed during 2007–2009 by active, community-based surveillance for febrile illnesses in a convenience sample of 32 rural villages in four districts of Kampong Cham province, Cambodia [23]. A total of 2359 paired sera samples collected from febrile patients were randomly selected among 7162 fever cases who tested negative for other infectious diseases (dengue, Chikungunya, Japanese Encephalitis, Influenza, Respiratory Syncitial virus and Human Metapneumovirus) [23]. A total of 100 cases showed a seroconversion indicative of an acute leptospirosis infection, and defined by a second serum sample positive for anti-*Leptospira* IgM (PanBio Leptospira IgM ELISA kit) while the first serum sample, collected ten days earlier during the acute febrile phase of the disease, tested negative [23]. Indeed, IgM are usually detectable in the blood around ten days after the onset of the disease; two sera samples are therefore necessary to confirm an acute leptospirosis. The database used in our study was a subset of the Hem et al [23] study and included only the 2359 cases who were IgM seronegative for the first serum sample collected. The variables retained from the original database are the information about the leptospirosis infection (serology results for leptospirosis and seroconversion status), information about the location of the villages (the villages code and geographical coordinates) and additional information about the patients that have been suggested as risk factors for leptospirosis infection (the age and the gender) [12,17,19].

### 2.2. Selection of the best remotely sensed flooding indicators

The remote sensing data used for water detection were MODIS TERRA MOD09A1 Surface-Reflectance Product, resolution 500m), from the Land Processes Distributed Active Archive Center of NASA (National Aeronautics and Space Administration) (http://e4ftl01.cr.usgs.gov/MOLT/MOD09A1.005/). These data are easily and freely accessible online and could therefore be used by local and national health authorities, even in countries with scarce resources dedicated to disease surveillance.

No water or flooding indicator had been tested to date in Cambodia to our knowledge. Six potential flooding indicators were selected based on the literature. Table 1 presents their characteristics.

**Table 1:** Summary of potential flooding indicators, their general formula, matched with MODIS band formula and their values.

Indicator	General Formula	MODIS Band Formula	Value range
NIR [33,34]	-	b01	Superior to 0
NDVI [35]	NDVI = $\frac{NIR-R}{NIR+R}$	(b01-b02)/(b01+b02)	Between -1 and 1
EVI [36]	EVI = $2.5\frac{NIR+R}{NIR+6R-7.5B+1}$	2.5((b01+b02)/(b01+6b02-7.5b03+1))	Between -1 and 1
NDWI [37]	NDWI = $\frac{G-NIR}{G+NIR}$	(b04-b01)/(b04+b01)	Between -1 and 1
NDII [38]	NDII = $\frac{NIR-MIR}{NIR+MIR}$	(b01-b06)/(b01+b06)	Between -1 and 1
MNDWI [39,40]	MNDWI = $\frac{G-MIR}{G+MIR}$	(b04-b06)/(b04+b06)	Between -1 and 1

Note: NIR = Near Infrared Red, NDVI = Normalized Difference Vegetation Index, EVI = Enhanced Vegetation Index, NDWI = Normalized Difference Water Index, NDII = Normalized Difference Infrared Index, MNDWI = Modified Normalized Difference Vegetation Index.

We had to choose the indicator best suited to the landscape studied. Cambodia land cover is characterized by vegetation (trees, plantations) and water (paddy fields).

**Fig 1:**
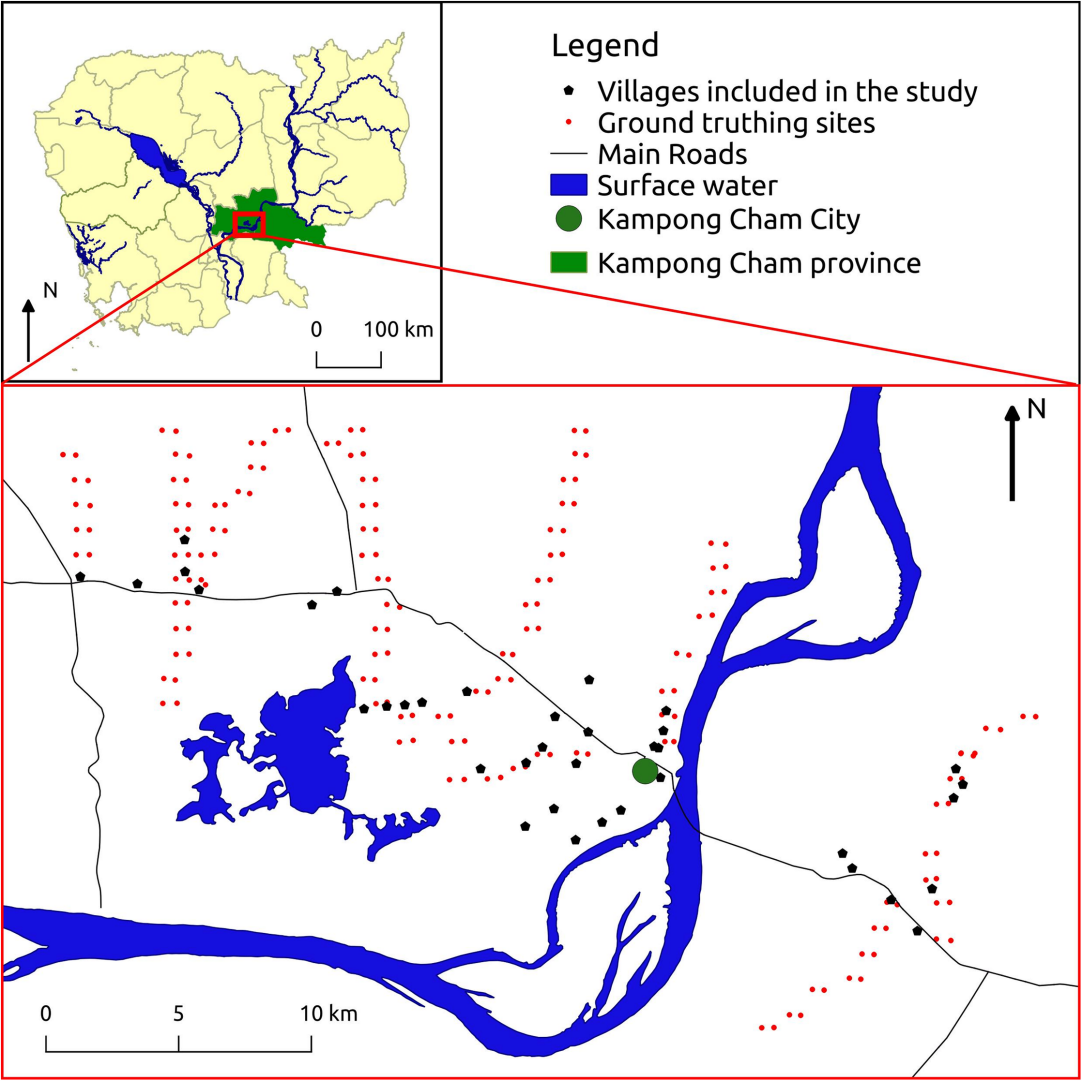
Study area and sites, Kampong Cham province, Cambodia (See S1 Table for the village names in English and Khmer). The main map is showing the locations of the villages included in the epidemiological study about human leptospirosis as well as the locations of the sites were the field data were collected for the ground truthing of the flooding indicator. The smaller map shows the location of the study area in Cambodia.

Remotely sensed flooding indicators are continuous variables and the range of values corresponding to water presence must be determined. One of the methods used to validate such indicators is to make observations in the field which are then used to calibrate the indicator for the specific area studied. These field observations were carried out in Kampong Cham province to match with the available leptospirosis data. A total of 230 locations were regularly observed along several road transects covering the study area (Fig 1).

Six observations were made every other week for each location between May and July 2014. The first observations were made on 05/05-05/07 and 05/19-05/21 during the dry season which lasts from December to May and the following during the beginning of the rainy season (06/02-06/03; 06/16-06/17; 07/08-07/09; 07/21-07/22). The observations consisted of observing and photographing the presence of water in the georeferenced square represented by the MODIS pixel. A ground truthing site was considered as flooded when at least one centimeter of water was covering more than half of the area of the corresponding MODIS pixel. But most of the time, the assessment of the presence of water was straightforward as highlighted by the pictures we added as S1 Fig.

For each observation, the corresponding MODIS image was downloaded and the six different flooding indicators were calculated.

The sensitivity and the specificity of the potential indicators were calculated for 40 thresholds ranging between the maximal and the minimal values of the indicators observed in the pixels studied. A receiver operating characteristic (ROC) curve was drawn, area under the ROC curve (AUC) was calculated and the optimal threshold was identified for each flooding indicator. The ROC curve is obtained by plotting the sensitivity depending on 1-specificity of the model. The AUC is an indicator of performance ranging from 0.5 (equivalent to a random test or model) to 1 (perfect test or model). The optimal threshold was the indicator value maximising both the sensitivity and the specificity of the water detection by the indicator.

The indicators with the best AUC were selected for the subsequent analyses.

The best flooding indicator and its optimal threshold for detecting water were then used to create a new variable: the number of weeks since the first flooding of the year. We added this new variable because it has been suggested that during the dry season, bacteria can be highly concentrated in the soil in limited area and that the first flooding may disseminate leptospira to more distant areas [32]. The first inundation of the year could then wash the soil where leptospira were concentrated during the dry season and may be more strongly associated to leptospirosis infections than subsequent flooding. In order to eliminate short term variations and catch the seasonal trend, the values of the flooding indicator used to determine the first flood of the year were smoothed over three weeks. The value for a given week was the mobile mean calculated over 3 weeks (W-1, W0 and W+1). The week of the first flooding was then defined as follows: the smoothed value of the indicator is greater than the optimal threshold and there is no smoothed values of the indicator are greater than the optimal threshold in the previous eight weeks.

### 2.3. Additional data

To evaluate the potential use of the best flooding indicator in predicting the distribution of human leptospirosis infections, we included additional data on population density and altitude, factors that may be related to leptospirosis infections [17,29]. For population density, we used a layer based on the 2008 national census that gave information with a 100m spatial resolution. For altitude, we used a Digital Elevation Model (DEM) with a 90m spatial resolution from the Shuttle Radar Topography Mission center on Cambodia developed by the Center for Spatial Information Science and Systems (George Mason University) and available on http://ws.csiss.gmu.edu/DEMExplorer/. For each fever case, altitude, population density and the flooding indicator were averaged spatially in a buffer of 1km around the center of the village. Each averaged value was then used in the GLM.

### 2.4. Flooding indicator and leptospirosis risk

Using the best flooding indicator, we tested whether that flooding indicator is also related to leptospirosis infections using univariate analysis and multivariate analysis, the latter to verify whether the link between leptospirosis and the detection of flooding by the indicator remained even when factors known to affect leptospirosis infections were taken into account.

First, we performed a chi-square test and estimated the rate ratio (RR) of leptospirosis infection associated with flooding detected through remote sensing. For each of the 2359 patients in the leptospirosis database, we estimated a flood variable by calculating the average value of the best flooding indicator for all the pixels included in a 1km radius around the village of the patient. If this average was above the optimal threshold, the village was considered as exposed to flooding; if not, the village was considered as not exposed to flooding. We performed chi-square tests and estimated the RR using the leptospirosis infection status and this flood variable. The RR divides the cumulative incidence in exposed group by the cumulative incidence in the unexposed group, determining whether being exposed to a factor is significantly associated with infection. The estimation of the RR was stratified on the season to ensure that the indicator was not only associated with the rainy season when flooding and most leptospirosis cases occur and was actually a relevant indicator within the rainy season. Thus, we also estimated the RR of leptospirosis infection associated with flooding detected through remote sensing during the rainy season only. A sensitivity-specificity analysis was also conducted on the best flooding indicator to estimate the optimal threshold of this indicator to discriminate leptospirosis infections and compare this threshold with the optimal threshold to discriminate between flooding and non-flooding areas. We used all the leptospirosis-confirmed cases (n = 100) and 300 randomly-selected non-leptospirosis cases. The same method was used as above.

Second, we used a generalized linear model (GLM) to explore the correlation between the best remotely-sensed indicator and leptospirosis infections while taking into account factors known to be related to leptospirosis infection. Stepwise descending logistical regression was performed to explore the risk of leptospirosis infection linked with flooding, after adjustment for other known risk factors for leptospirosis: adult age [12,17,19], male gender [12,17,19], rural setting [17], altitude [29] and rainfall [28]. A stepwise descending procedure starts with the full model (the model including all the explanatory variables) and tries to remove explanatory variables at each step to simplify the model. An explanatory variable is removed when the evaluation criterion of the resulting model is improved. We used the Akaike information criterion (AIC) which takes into account the maximum likelihood and the parsimony of the model. We then calculated the Area Under the Curve (AUC) on trained data (TD AUC, estimated from the same data used to train the model) and by cross-validation (CV AUC, estimated from 75% of the data, the other 25% being used to train the model) to assess the performance of the model.

In order to take a deeper look at the association between leptospirosis infection and explanatory variables and in particular flooding, we also conducted Boosted Regression Tree modeling (BRT). BRT is a machine-learning method based on two algorithms: multivariable regression trees and a boosting process that combines simple models to improve performance. The BRT method was selected because it provides better predictions than generalised models and is able to deal with complex responses such as non-linear relationships and interactions between variables [41]. BRT assesses the relative importance (RI) of each explanatory variable, based on the number of times a variable is used in all trees and its contribution to the final model improvement.

The R software (version 3.1.0; The R Foundation for Statistical Computing, Vienna, Austria)[42] packages dismo (version 0.9–3) [43] and gbm (version 2.1) [44] were used to implement the BRT model. We used a tree complexity of 5, a learning rate of 0.0001, a bag fraction of 0.5 and the number of trees was optimized using the step.gbm function.

In order to estimate the performance of the BRT model, we estimated the average trained data AUC (TD-AUC, estimated from the same data used to train the model) and Cross-Validation AUC (CV_AUC, estimated from half of the data, the other half being used to train the model) and the standard deviations were calculated over 10 iterations. These parameters provide the percentage of variability explained by the model in the data used to build the model and with bag fraction data not used to build the model, respectively.

#### Ethical considerations

The leptospirosis data were analysed anonymously. The leptospirosis database was obtained from a previous study approved by the National Ethic Committee for Health Research in Cambodia NECHR on April8^th^, 2011(#NEHCR35-2011).

## 3. Results

### 3.1. Selection of the best remotely sensed flooding indicators

**Fig 2:**
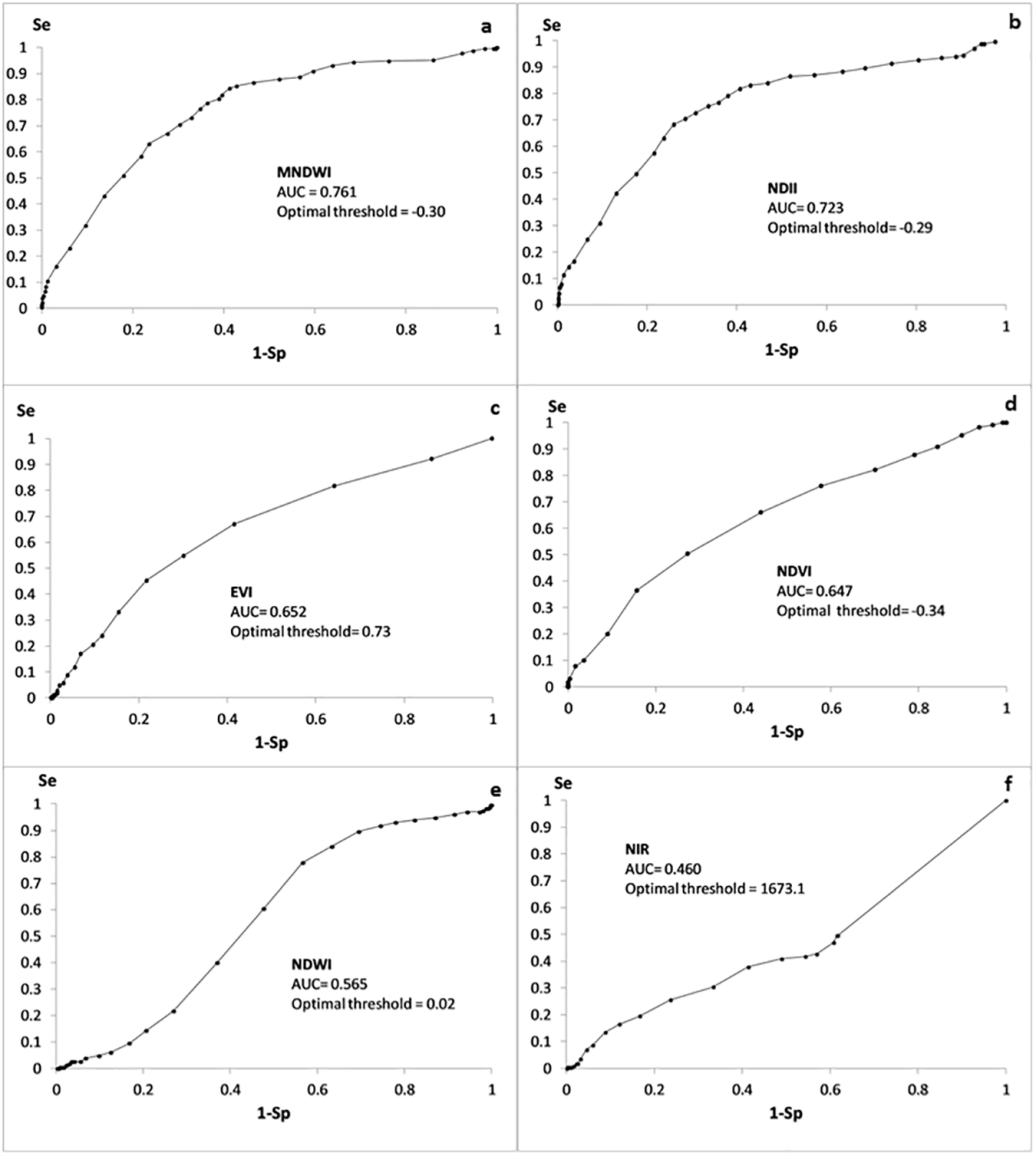
ROC curves for a EVI; b NDWI; c NIR; d MNDWI; e NDII; f NDVI, n = 1217, May-July 2014, Kampong Cham, Cambodia.

Water presence was documented at 230 locations in May-July 2014 but only 152 of these locations remained accessible during the rainy season. A total of 1,217 MODIS pixels were documented to calibrate the indicator values. The ROC curves for each potential flooding indicator are shown in Fig 2. MNDWI had the best AUC with 0.761 and was selected as the flooding indicator and used in the following analysis.

### 3.2. Flooding indicator and leptospirosis risk

Results from the univariate analyses showed a statistically significant correlation between leptospirosis infections and areas considered as exposed to flooding (based on averaged MNDWI values superior to -0.3). The Risk Ratio was 1.61 [1.10–1.52] and the chi-square test value was 5.64, (p-value = 0.02) for the whole dataset. The correlation was even stronger during the rainy season only (RR = 2.03 [1.25–3.28], chi^2^ = 8.15, p-value = 0.004). During the rainy season, people exposed to floods had a twice greater risk of being infected by leptospirosis.

The optimal threshold for discriminating leptospirosis cases with MNDWI values was the same that the one to discriminate flooded areas, -0.3.

The best GLM model identified by the stepwise descending process included age, altitude and the time elapsed since the first flooding of the year (Table 2). It had a training data AUC of 0.616 and a cross-validation AUC of 0.558.

**Table 2:** Summary of the regression analysis to explain leptospirosis cases in Kampong Cham Province, Cambodia, 2007–2009, n = 1832.

Variable	coefficient	CI95%	p-value
*Best model*:			
intercept	-3.382	[-4.420; -2.405]	4.6e-11
Age	0.054	[0.013; 0.095]	0.009**
Altitude	0.011	[-0.017; 0.040]	0.437
Time since first flood	-0.015	[-0.029; -0.001]	0.042*

* indicate significance at 95% and

** indicate significance at 99%.

The BRT model had an average training data AUC of 0.80 (SD = 0.002) and an average CV AUC of 0.572 (SD = 0.009). The final model had 10080 trees, a learning rate of 0.0001, a tree complexity of 5 and a bag fraction of 0.5.

MNDWI was the variable that contributed the most to the model (relative influence of 26%), followed by altitude (23%) and time since first flooding (19%).

**Fig 3:**
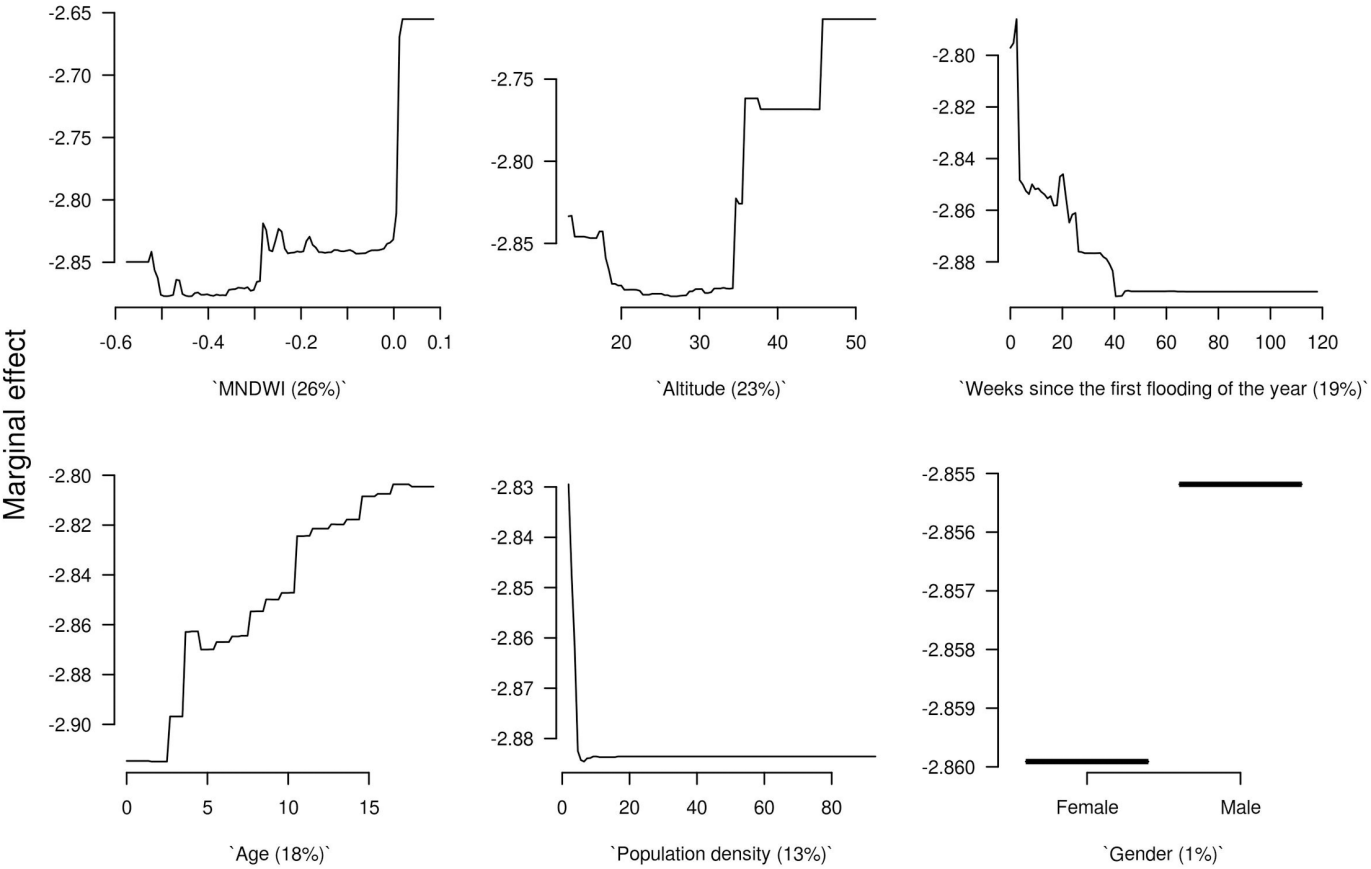
Marginal effect curves of each explanatory variable of the BRT model. The sub-plots are ordered by the mean of their relative influence to the BRT model, with these RI given in parentheses with each sub-plot.

The effect of each explanatory variable on the response variable is shown in Fig 3. The MNDWI indicator had two thresholds; the risk for leptospirosis infection was higher for MNDWI values greater than -0.3) (Fig 3A) and increased again for MNDWI values greater than 0. The risk was higher just after the first flooding of the year and decreased slowly until 40 weeks after the first flooding (Fig 3C). Children aged below four and people living in villages with an altitude below 35 meters had a lower risk of seroconversion (Fig 3D and 3B). Maps of the MNDWI in the study area during rainy (July 2009) and dry (April 2009) seasons are presented in S2 Fig.

## 4. Discussion

In this study—the first of its kind to our knowledge in Cambodia—we (i) assessed the performance of six different remotely-sensed flooding indicators and (ii) assessed whether the most effective one could be used in predicting the distribution of human leptospirosis infections at local level in Kampong Cham province, Cambodia. MNDWI was the best flooding indicator based on field observations. Interestingly, the threshold of -0.3 maximizing the performance of MNDWI as a flooding indicator was also the optimal threshold to discriminate leptospirosis infection according to both our sensitivity-specificity evaluation methods and the BRT model. Two independent analyses based on independent datasets showed that MNDWI values greater than -0.3 were significantly associated with both flooding and an increased risk of leptospirosis infection even during the rainy season (RR = 2.03 [1.25–3.28], chi-square = 8.15, p-value = 0.004). This is consistent with what is known of leptospirosis epidemiology and the role of flooding in leptospirosis outbreaks [13,18,20,31,45–47] but differs from the results of Suwanpakdee et al. which did not find a direct correlation between leptospirosis cases and flooding in Thailand [48]. However, they used confirmed and suspected leptospirosis cases reported to the National surveillance system—that may lead to an important under-detection of the real number of cases- whereas we used confirmed cases from a community active surveillance of undifferentiated fevers [23,48]. Our results illustrate the potential for this flooding indicator to be used as an early warning predictor of an increased leptospirosis risk in Cambodia and probably in other countries for leptospirosis or for other diseases strongly associated with flooding.

In addition to detecting flooded areas using MNDWI, we also included the time elapsed since the first flooding of the year in our GLM and BRT models. This variable, estimated using MNDWI, was a significant explanatory variable in the GLM (p = 0.042) and had an important influence in the BRT model (relative influence of 19%).This is consistent with a Brazilian study that observed peaks of leptospirosis cases three to five weeks after floods in Rio De Janeiro [32]. This importance of the first flooding of the year may be explained by the dissemination of high concentration of Leptospira bacteria accumulated locally during dry season [13,30,32]. Indeed, flooding and heavy rainfall cause increased runoff and washing of fecal material and Leptospira into the environment, including bathing and drinking water [8,13,32].The BRT model suggests that the risk of leptospirosis infection is higher within the ten weeks after the first flooding of the year and slowly decreases until the next rainy season. If this is confirmed early warning epidemiological tools based on remotely-sensed indicators could inform on, quantify and monitor the first flooding of the year and guide timely and targeted public health action.

Our models (GLM and BRTs) should not be seen as tools to accurately predict leptospirosis risks in Cambodia at this stage. Several limitations could explain the low performance of the models assessed by cross-validation (AUC = 0.558 for GLM and 0.572 for BRT) despite a good performance of the BRT model on the training data (AUC = 0.8). The leptospirosis data we used were a partial byproduct of a study designed to study dengue [23]. The dataset we used was not initially designed to undertake a spatial analysis of leptospirosis seroconversions. The fever cases were not independent because children came from a limited number of villages. Furthermore, one child could have several fever episodes during the study, potentially leading to multiple inclusions in the initial study. Finally, the main limitation is the limited study area of 42 km by 25 km in a single province of Cambodia.

In our study, we used an indicator of water presence/absence and did not take into account the different classes of water such as surface water, waterlogged soils, flooded vegetation nor did we take into account other information on the water such as temperature, salinity, turbidity or depth that can have an impact on pathogen transmission [10]. As reviewed by Tran et al [10], using different sensors can help to discriminate between different water classes, radar remote sensing being potentially better at identifying several types of inundated land. Additional information about water is difficult to collect through remote sensing only [10]. Depth of flooding may have played a role in our study because it can have an impact on bacteria dissemination and titers which determine the risk of infection for humans, with an increased risk of infection for shallower inundations as observed in Cambodia for coliform bacteria [49]. As already discussed, the results of our BRT model suggesting that the first flood of the year is associated with an increased risk of leptospirosis may be related to this difference of bacteria concentration in shallower water. This parameter could be taken into account by developing a hydrologic model as done by Kazama et al [49].

In Senegal, Soti el al [50] combined remote sensing and hydrologic modeling to assess the spatio-temporal dynamics of ponds in the Ferlo Region to better understand the epidemiology of Rift Valley Fever, a vector-borne disease. Mechanistic models taking into account hydrologic connectivities are better at mapping the dispersion of pathogens with water flow. Remais et al [51] improved the predictive power of their schistosomiasis transmission model in China by including hydrological data. A spatially explicit model of cholera transmission developed by Bertuzzo et al [52] helped understand the factors driving the annual dual-peak of cholera observed in Bangladesh. Developing such mechanistic approaches or using different sensors to discriminate between water classes would certainly improve our model, our knowledge and our capacity to forecast waterborne diseases. It would, however, increase the complexity of any early warning tool based on a model and consequently hinder its implementation by health authorities. As highlighted by Lleo et al [8], the use of remote sensing data in health applications could be better promoted and a simple statistical model improving risk forecasting could be a first step for health specialists unfamiliar with this technology. The development of a hydrologic model to better understand the epidemiology of leptospirosis could then lay the groundwork for further research work aiming at identifying the main drivers of leptospirosis in Cambodia.

As highlighted by Hamm et al., the quality of remote sensing data needs to be assessed as some parameters such as clouds can affect quality of the imaging [53]. The range of values corresponding to a specific element could also be affected and may be time and space-dependent. It was therefore important to identify the range of values corresponding to the detection of water bodies on the ground and to assess the sensitivity and specificity of the MNDWI indicator to detect water in our area [54]. The MODIS images used are aggregated on eight days and data quality is assessed and maximized, using correction for atmospheric gases and aerosols and selection of the best pixels [55]. Further corrections could be made on these remote sensing data to increase their quality, but such additional technical work would certainly prevent routine users like local and national health authorities to use them as an early warning tool. Given the objective of this study, and since we are not exploring the mechanisms relating flooding to leptospirosis, we did not use further corrections of the remote sensing data.

Having the flooding indicator as the most influent explanatory variable in the BRT and GLM models in a small and homogenous study area is encouraging and one can expect an improved performance of the indicator and of the models, when dealing with more contrasted areas. Furthermore, a specifically designed study allowing for the use of other explanatory variables at a larger scale should increase the performance of the models.

If such improvements were possible, the use of MNDWI could help target risk areas to design prevention plans or inform clinicians in a timely way of a locally increased risk of leptospirosis, a potentially lethal disease which requires specific treatment. The MNDWI and remotely-sensed indicators in general provide information on a very large scale. Satellite imaging is freely and quickly available, and could contribute to an early-warning system based on quasi real-time risk prediction. Health workers could thereby be informed of the higher proportion of children expected to be infected by flood-related diseases in the subsequent weeks. This could help planning and stock-piling of appropriate antibiotics for leptospirosis treatment and better patient diagnosis and management. Such a surveillance and early warning system could be most effective and useful for health workers if associated with surveillance for dengue, the first differential diagnose of leptospirosis in tropical countries where both pathogens circulate.

In conclusion, our study, aimed solely at assessing the performance of remotely-sensed flooding indicators as risk indicators of flood-driven diseases, found a significant correlation between leptospirosis and MNDWI as a proxy for flooding, even when using rainy season data. Even though this correlation does not inform us on the underlying mechanisms and the potential causal links, it can still be used practically to assist in epidemiological surveillance and to provide early warning in a timely and adequate way. Despite limitations preventing us from using our model for leptospirosis prediction at this early stage, our study suggests the potential usefulness of flooding indicators and—of MNDWI in particular—for the analysis and the development of tools to better predict the risk of leptospirosis and potentially other flood driven diseases in Cambodia and other Southeast Asia settings.

## Supporting information

S1 Table The village names in English and Khmer. (DOCX)

S1 Fig Pictures successively taken at one site to support the ground truthing of water presence, Kampong Cham Province, Kingdom of Cambodia, May-July 2014. (TIF)

S2 Fig MNDWI values in the study area represented continuously during the dry (a) and the rainy (b) season and represented as a discrete variable using the threshold -0.3 during dry (c) and rainy (d) season in 2009, Cambodia.
(TIF)
