# Supplementary material for: Assessing the performance of remotely-sensed flooding indicators and their potential contribution to early warning for leptospirosis in Cambodia
Source: PLoS One. 2017 Jul 13;12(7):e0181044. doi: 10.1371/journal.pone.0181044 (PMC5509259; doi:10.1371/journal.pone.0181044)
Supplement: S1 Table — (DOCX) [file pone.0181044.s001.docx]

Supplementary Table 1: Village code, initial and names in Khmer and English

| Village Code | Village Name Latin | Village Name Khmer | Village Initials |
| --- | --- | --- | --- |
| 3050101 | Boeng Kok Muoy | bwgkuk 1 | BM |
| 3050103 | Chong Thnal Muoy | cugfñl; 1 | CM |
| 3050104 | Chong Thnal Pir | cugfñl; 2 | CP |
| 3050107 | Memay | emma:y | MM |
| 3050202 | Phum Prambei | PUmi 8 | PB |
| 3050302 | Roka Kraom | rkaeRkam | RK |
| 3050304 | Boeng Basak | bwg)asak; | BB |
| 3050307 | Preaek Chan | ERBkcan; | PC |
| 3050308 | Preaek Chik | ERBkCIk | PH |
| 3050309 | Kampong Roling | kMBg;rlIg | KL |
| 3060102 | Ampil Leu | GMBilelI | AL |
| 3060103 | Ampil Kraom | GMBileRkam | AK |
| 3060106 | Romeas | rmas | RM |
| 3060107 | Banteay Thmei | bnÞayfµI | BH |
| 3060108 | Sralau | RseLA | SL |
| 3060109 | Krala | RkLa | KR |
| 3060111 | Veal Sbov | vals,Úv | VS |
| 3060112 | Sya | süa | SY |
| 3061501 | Prey Phdau | éRBepþA | PP |
| 3061502 | Andoung Svay | GNþÚgsVay | AS |
| 3061503 | Kouk Kream | eKakRKam | KK |
| 3061504 | Kdei Boeng | kþIbwg | KD |
| 3130810 | Krasang Pul | RksaMgBul | KP |
| 3130819 | Traeung | eRtIg | TG |
| 3131502 | Pring Bei Daeum | RBIgbIedIm | PD |
| 3131503 | Chachak | cck | CH |
| 3131510 | Ou Da | GUrda | OD |
| 3131512 | Tuol Ampil | TYlGMBil | TA |
| 3131519 | Kakaoh | kekaH | KH |
| 3160601 | Tuol Vihear | TYlvihar | TV |
| 3160602 | Srae Siem | ERsesom | SS |
| 3160603 | Tuek Chenh | Twkecj | TC |
| 3160605 | Tuol Ponley | TYlBénø | TP |
| 3160607 | Andoung Chea | GNþÚgCa | AC |
| 3160608 | Ta Trav | taRtav | TT |
| 3160609 | Chruoy Kor | RCYyKr | CK |
| 3160610 | Boeng Tras | bwgRts; | BT |
