# Supplementary figures and images for: Assessing the performance of remotely-sensed flooding indicators and their potential contribution to early warning for leptospirosis in Cambodia
Source: PLoS One. 2017 Jul 13;12(7):e0181044. doi: 10.1371/journal.pone.0181044 (PMC5509259; doi:10.1371/journal.pone.0181044)

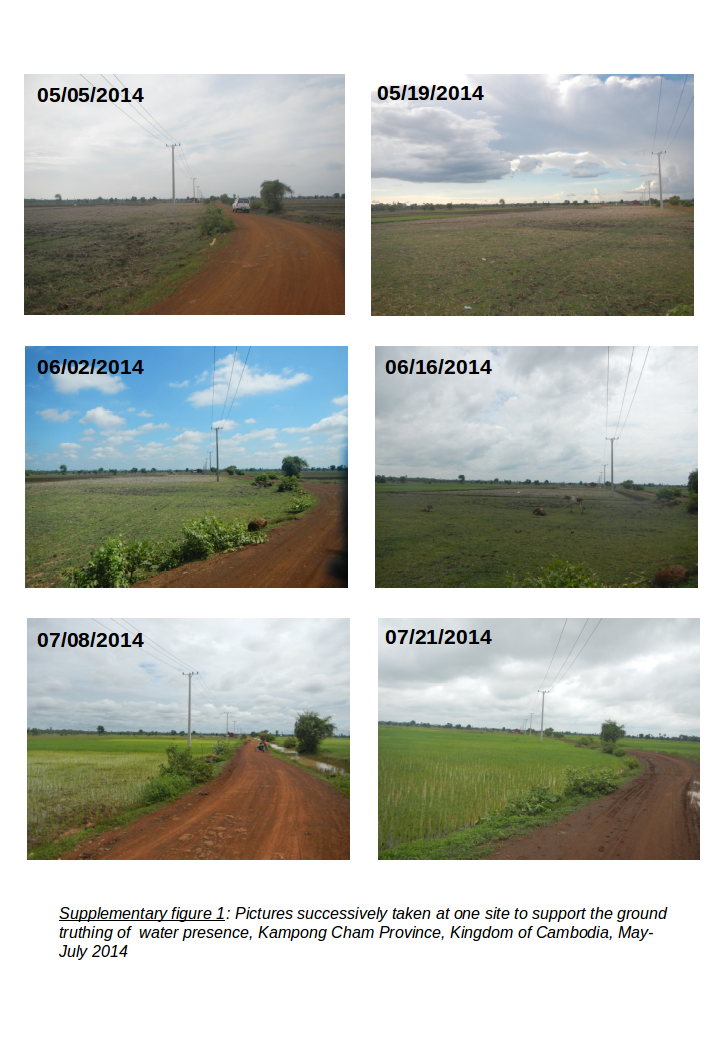

Supplement: S1 Fig — (TIF) [file pone.0181044.s002.tif]

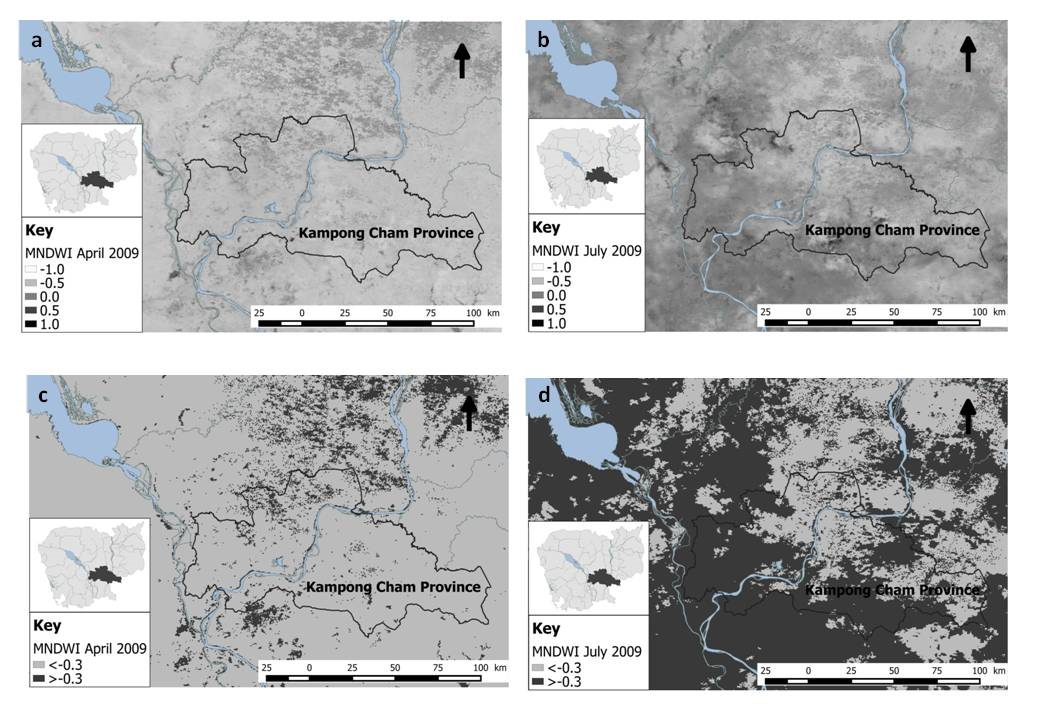

Supplement: S2 Fig — MNDWI values in the study area represented continuously during the dry (a) and the rainy (b) season and represented as a discrete variable using the threshold -0.3 during dry (c) and rainy (d) season in 2009, Cambodia. (TIF) [file pone.0181044.s003.tif]
